# Hybrid *Bis*-(Imidazole/Benzimidazole)-Pyridine Derivatives with Antifungal Activity of Potential Interest in Medicine and Agriculture via Improved Efficiency Methods

**DOI:** 10.3390/ph18040495

**Published:** 2025-03-28

**Authors:** Tiberius Balaes, Violeta Mangalagiu, Vasilichia Antoci, Dorina Amariucai-Mantu, Dumitrela Diaconu, Ionel I. Mangalagiu

**Affiliations:** 1 Department of Biology, Faculty of Biology, Alexandru Ioan Cuza University of Iasi, 20A Carol 1st Bvd, 700505 Iasi, Romania; tiberius.balaes@gmail.cm; 2 Institute of Interdisciplinary Research, CERNESIM Centre, Alexandru Ioan Cuza University of Iasi, Bd. Carol I, No. 11, 700506 Iasi, Romania; violeta.mangalagiu@uaic.ro; 3 Faculty of Food Engineering, Stefan Cel Mare University of Suceava, 13 Universitatii Str., 720229 Suceava, Romania; 4 Faculty of Chemistry, Alexandru Ioan Cuza University of Iasi, Bd. Carol I, No. 11, 700506 Iasi, Romania; vasilichia.antoci@uaic.ro (V.A.); dorina.mantu@uaic.ro (D.A.-M.); 5 Institute of Interdisciplinary Research, RECENT-AIR Centre, Alexandru Ioan Cuza University of Iasi, Bd. Carol I, No. 11, 700506 Iasi, Romania

**Keywords:** imidazole, benzimidazole, pyridine, hybrid *bis*-(imidazole/benzimidazole)-pyridine, human fungi, phytopathogenic fungi, antifungal, medicine, agriculture, ultrasounds

## Abstract

**Background/Objectives**: Nowadays fungal infections are rising serious threats for the human health system and agriculture, mostly because of antifungal resistance, emergence of new fungal pathogens and adverse effects, pressing the scientific world for exploration of new antifungal compounds. Therefore, the aim of this work was to synthesize and to study antifungal activity against human and plant fungi of a new class of hybrid *bis*-(imidazole/benzimidazole)-pyridine salt derivatives. **Methods**: The synthesis of the hybrid derivatives was performed using both conventional thermal heating and ultrasound irradiation methods. **Results**: The use of ultrasound irradiation has the advantages of a dramatic decrease in reaction time and, consequently, a notable acceleration in reaction rate, a remarkable decrease in consumed energy and higher yields. The antifungal activity against five human fungal strains and for plant fungal strains was determined by the disk diffusion method and minimum inhibitory concentration. **Conclusions**: The tested hybrid derivatives manifest good antifungal activity against the tested strains. Some of the hybrid compounds have very good quasi-nonselective activity against the tested human and plant pathogenic fungi, in some cases close to the control drug fluconazole, respectively, to many antifungal agents commercially used for plant protection.

## 1. Introduction

**Figure 1:**
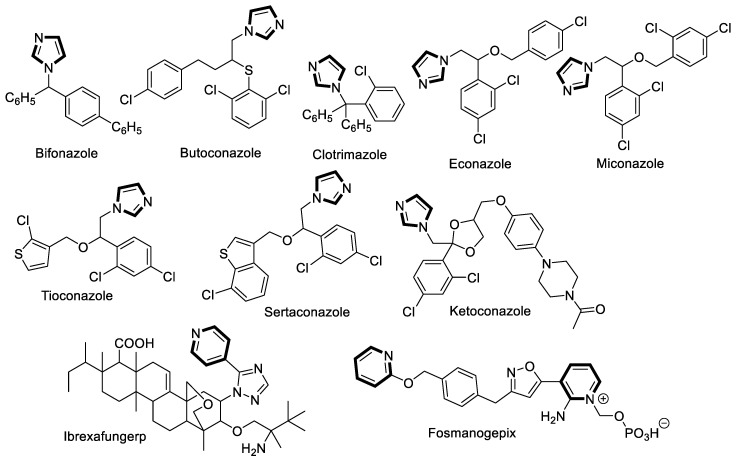
Antifungal drugs with imidazole and pyridine skeleton frequently used in current human therapy.

During the last decades fungal diseases have raised serious concerns for human health systems and agriculture (for crop protection) [1,2,3,4], mostly because of the antimycotic resistance phenomena caused by long-term use and misuse of the existing antifungal drugs and also because of the emergence of new fungal pathogens [5,6,7,8]. Recently [8], the World Health Organization (WHO) categorized invasive fungal infections as diseases of great risk for humans, with fungal pathogens being ranked into three categories of risk: medium (this includes *Coccidioides* spp., *Candida krusei*, *Cryptococcus gattii*, *Lomentospora prolificans*, *Pneumocystis jirovecii*, *Paracoccidioides* spp., *Scedosporium* spp. and *Talaromyces marneffei*), high (this includes *Candida glabrata*, *Candida parapsilosis*, *Candida tropicalis*, *Fusarium* spp., *Histoplasma* spp. and eumycetoma causative agents, Mucorales) and critical (this includes *Cryptococcus neoformans*, *Candida auris*, *Aspergillus fumigatus,* and *Candida albicans*). According to the literature data [1,2,9,10,11,12], the armamentarium used to combat fungal diseases includes four classes of drugs: allylamines, azoles, echinocandins, polyenes, and calcium-calcineurin derivatives (to some extent, there is a 5th one, the antimetabolites, which are pyrimidine derivatives), with each of them boasting its own mechanism of antifungal activity. Among them, the azole classis is the most used and representative from the market, with drugs containing imidazole and triazole azaheterocycles being the most important and efficient category of drugs used in current antifungal therapy [1,13,14]. In Figure 1 are listed some of the most representative clinically used drugs with imidazole skeleton, these include *bifonazole*, *butoconazole*, *clotrimazole*, *econazole*, *miconazole*, *tioconazole*, *sertaconazole*, *ketoconazole*, etc. [1,12,13,14,15,16].

Recently research data indicate that azine derivatives, especially those bearing azole units, have a great antifungal potential [1,15,16], with some of these compounds already being drugs on the market, Figure 1. Thus, *fosmanogepix* and *ibrexafungerp* (Figure 1) are antifungal drugs with a pyridine skeleton (bearing oxazole or triazole units) [15,16,17]. However, the number of existing drugs from the market (or existing in clinical trials) from this class is very low.

**Figure 2:**
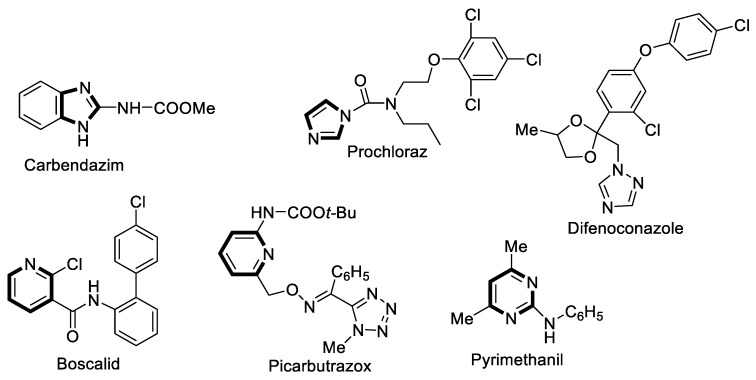
Antifungal pesticides with azole and azine skeleton frequently used in agriculture.

On the other hand, pathogenic fungi are a serious threat in agriculture for the growth of crops (especially cereals, fruits and vegetables), producing great economic losses worldwide every year. During the last period, the main approaches to assure the protection of crops against pathogenic fungi involve the use of pesticides, particularly those with azole and azine heterocycles skeletons, such as *carbendazim*, *prochloraz*, *difenoconazole*, *boscalid*, *picarbutrazox*, *pyrimethanil*, etc. [18,19], Figure 2.

Because of the excessive and unreasonable use of existing antifungal pesticides, many fungal strains gain resistance causing supplementary problems, especially economic ones [18,19]. Despite the recent advances in the class of azole and/or azine derivatives with antifungal properties, discovering new antifungal agents with better antifungal properties remains a main goal for scientists.

The literature describes many methods of synthesis for antifungal compounds with azole and azine skeletons, using conventional or nonconventional methods [20,21,22,23,24,25]. Over the last few years, ultrasound (US) irradiation has become a very useful tool in organic and medicinal chemistry, emerging as an extremely useful tool for a large variety of syntheses [26,27,28]. Compared with conventional thermal heating (TH) methods, US irradiation provides some undeniable advantages in organic synthesis. These include reduced reaction times, energy saving, use of smaller amounts of solvents, lesser or no side reactions, better yields, higher purities of products, the possibility to reach new selectivity and new reactivity in chemical reactions, many times cheaper costs, and ease of handling and processing [29,30,31,32,33,34]. As a result, reactions under US irradiation become environmentally friendly [29,30,31,32,33,34].

Motivated by the above considerations and having in view our previous expertise in the field of US-assisted reactions [35,36,37], as well as in imidazole and pyridine azaheterocycles with antifungal activity [38,39,40,41,42], this study aimed to obtain and determine the antifungal activity against human and plant fungi of a new class of hybrid *bis*-(imidazole/benzimidazole)-pyridine salt derivatives. In equal measure, we were interested in performing the synthesis of these hybrid compounds by using environmentally friendly methods under US irradiation.

## 2. Results and Discussions

### 2.1. Design, Mechanism of Action and Synthesis

**Scheme 1:**
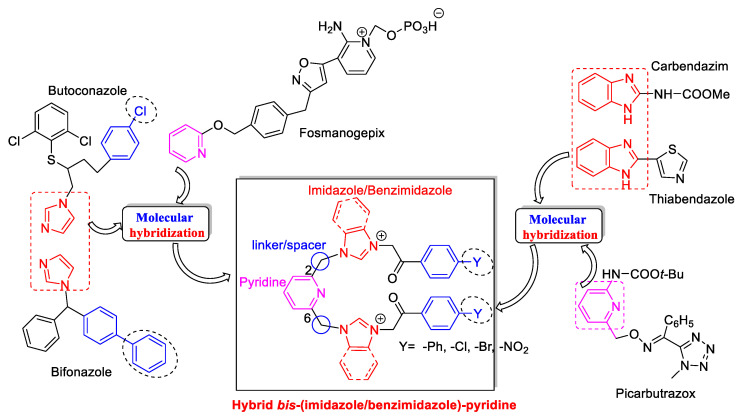
Design in the class of hybrid *bis*-(imidazole/benzimidazole)-pyridine derivatives.

In the first instance, by using the molecular hybridization approach [40,42], we designed the new class of hybrid *bis*-(imidazole/benzimidazole)-pyridine derivatives by combining the pharmacophoric potential of the imidazole/benzimidazole azole moiety and the pyridine azine moiety, Scheme 1.

In this respect, the first nitrogen atom from the imidazole/benzimidazole pharmacophore unit was connected with the pyridine pharmacophore unit via a methylene (-CH_2−_) linker at 2,6-position of pyridine moiety, leading to the desired *bis*-(imidazole/benzimidazole)-pyridine hybrids. Having in view that the existing imidazole drugs from the market usually have linked to the second nitrogen atom from an imidazole moiety a *para*-Y-phenyl graph, we decided to introduce into our hybrid structures such a substituent and to study the influence against antifungal activity of this graph, by using bioisosterism lead modifications as Y-substituents, from the *para* position of benzoyl moiety were chosen the -Cl, -Br, -NO_2_ and -C_6_H_5_ (phenyl, -Ph) moieties. Also, being salts, the solubility in water of our compounds will increase, and we expect a serious improvement of pharmacokinetic properties, especially absorption.

As to the mechanism of antifungal action, it is well documented that imidazole derivatives are antifungal agents that block fungal sterol/ergosterol biosynthesis by inhibiting lanosterol 14 α-demethylase enzyme CYP51 [1,2] while pyridine drugs are cell wall inhibitors [15,16,17]. Moreover, the binding site of CYP51 contains a heme cofactor that can coordinate with an imidazole or triazole ring of azole drugs. Having in view these considerations, we are expecting that our hybrid compounds will act as multitarget ligands that could inhibit the CYP51 enzyme and produce inhibition of cell wall synthesis.

**Scheme 2:**
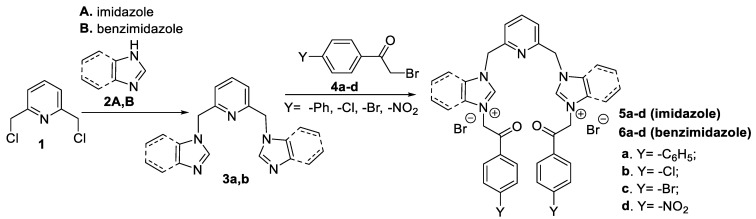
Synthesis of hybrid *bis*-(imidazole/benzimidazole)-pyridine salts derivatives **5a**–**d** and **6a**–**d**.

To synthesize our hybrid *bis*-(imidazole/benzimidazole)-pyridine derivatives, we used an adaptation of a previous method described by us [40,42], which involves a direct and efficient two-step reaction pathway, Scheme 2. In the first step, 2,6-*bis*(chloromethyl)pyridine **1** reacts with imidazole (**2a**)/benzimidazole (**2b**) via an *N*^1^-alkylation reaction, leading to the hybrid *bis*-(imidazole/benzimidazole)-pyridine **3a**,**b**. In the second step, compounds **3a**,**b** suffer a quaternization reaction of the second *N*^3^-nitrogen atom from imidazole/benzimidazole unit with α–bromo-*para*-Y-substituted-acetophenones **4a**–**d**, leading to the desired final products, the hybrid *bis*-(imidazole)-pyridine salts **5a**–**d** and hybrid *bis*-(benzimidazole)-pyridine salts **6a**–**d**.

Under conventional TH, the *N*-alkylation reactions have some major drawbacks, such as long reaction time (480–720 min), high amounts of consumed energy, moderate to good yields (around 45–85%), and large amounts of used solvents. Having in view these considerations and applying the principle of green chemistry, we decided to study these reactions by using US technology. The results obtained are presented in Table 1.

The data listed in Table 1 reveal that the use of US irradiation in the studied *N*-alkylation reactions has some certain advantages in terms of a dramatic decrease in reaction time (by four to seven folds) and, consequently, a notable acceleration of reaction rate, a remarkable decrease in the consumed energy, and higher yields (with about 10–15%). Unfortunately, the amount of solvent used was not possible to be decreased.

**Figure 3:**
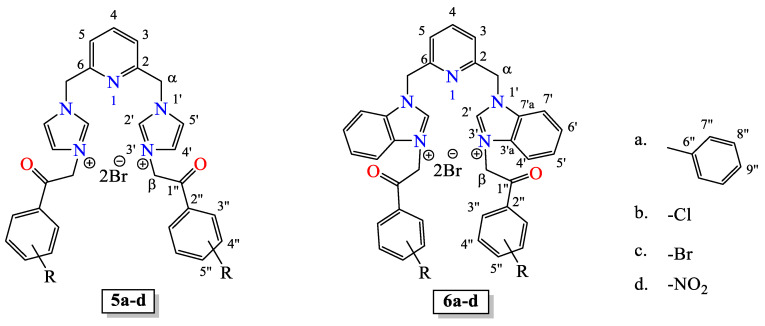
The numbering of atoms from hybrid *bis*-(imidazole/benzimidazole)-pyridine derivatives **5a**–**d** and **6a**–**d**.

The structures of the hybrid compounds **5a**–**d** and **6a**–**d** were proven by elemental (C, H, N) and spectral analysis: IR, ^1^H-NMR and ^13^C-NMR. If we consider compound **5a** as representative for the series, the most informative signals furnished by ^1^H-NMR spectrum are those of the aliphatic methylene protons (2H from CH_2-α_ and 2H from CH_2-β_, respectively), and the aromatic protons H_2′_ (from imidazole ring), H_2″_ and H_3″_ (from *para*-phenyl ring). The proton signals from the two methylene groups appear as singlet, at a very high chemical shift, unusual for this type of proton: 6.16 ppm (H from CH_2-β_) and 5.73 ppm (H from CH_2-α_). This displacement to high chemical shift is due to the powerful electron withdrawing effect of the adjacent positive nitrogen atom *N*^3′^ from the imidazole moiety and carbonyl group (in the case of CH_2_ from β position), respectively, nitrogen atom *N*^1′^ from imidazole and α-pyridine ring (in the case of CH_2_ from α position). The imidazole protons H_2_^′^ are the most unshielded from the spectra, being situated at 9.25 ppm (singlet), due to the powerful unshielded effect of the positive nitrogen atom *N*^3′^ and the strong electron withdrawing effect of *N*^1′^ nitrogen from imidazole ring. The next unshielded protons are H_2″_ from the phenyl ring moiety (8.11–8.09 ppm, doublet, J = 8.4 Hz), this proton being strongly unshielded by the electron withdrawing effect of the adjacent carbonyl ketone group (protons are in *orto* position relative to ketone functionality) and phenyl moiety (Y substituent) from the *para*-position of benzoyl moiety. The H_2″_ protons are coupled with H_3″_ (8.05–8.03 ppm, doublet, J = 8.4 Hz), also at high chemical shifts, for the same reasons as in the case of H_2″_ protons. In the ^13^C-NMR spectrum, the most informative data are furnished by the signals corresponding to carbon atoms of the carbonyl group, C_1″_ and C_4″_ (from phenyl ring moiety) and the two aliphatic carbons from the methylene groups (C from CH_2-α_ and C from CH_2-β_). The signal of the most unshielded carbon (from carbonyl ketone group) appears at 190.5 ppm, typical for a C=O carbonyl alkyl-aryl ketone group. The next unshielded carbons are C_1″_ and C_4″_ (from phenyl ring moiety) due to the powerful electron withdrawing effect of the carbonyl ketone and phenyl moiety (Y substituent) from the *para*-position of benzoyl moiety. Thus, C_1″_ carbon appears at a very high chemical shift (153.6 ppm), being in *ipso* position relative to ketone functionality and *para* position relative to phenyl moiety (Y substituent). The C_4″_ carbon appear also at very high chemical shifts (145.7 ppm), being in *para* position relative to ketone functionality and *ipso* position relative to phenyl moiety (Y substituent). The aliphatic carbons from methylene groups appear at a very high chemical shift, unusual for this type of carbon: 55.6 ppm (carbon from CH_2-β_), 52.7 ppm (carbon from CH_2-α_). The same explications for such a high chemical shift displacement of this aliphatic methylene carbons given in the case of protons remain valid in the case of carbons. All the remaining signals from NMR spectra are in accordance with the proposed structures. See also Supplementary Materials for the ^1^H- and ^13^C-NMR spectra of compounds and Figure 3 for atom numbering.

### 2.2. Antifungal Results

The *in vitro* antifungal activity against human and plant fungi of the hybrid *bis*-(imidazole/benzimidazole)-pyridine salt derivatives **5a**–**d** and **6a**–**d** was evaluated by the Kirby–Bauer disk diffusion method [43,44,45], using a Sabouraud nutrient agar medium for antifungal assay. The antifungal activity was determined against five fungal strains of medical importance (*Rhodotorula* sp., *Candida albicans* wild type, *C. parapsilosis* wild type—strains kindly offered by Dr. Simona Matiut from the Praxis Clinical Laboratory, Iasi, Romania, obtained from biological specimens; *C. albicans* ATCC 10231, *C. parapsilosis* ATCC 22019) and against four filamentous fungal strains of agriculture importance, from the culture collection of Fungal Research Laboratory, Faculty of Biology, Alexandru Ioan Cuza University of Iasi, isolated from fungal attack on cultivated plants (*Aspergillus niger* wild type, *A. flavus* wild type, *Cladosporium cladosporioides* wild type, *Rhizopus nigricans* wild type).

In Table 2 are listed the results of the antifungal assay determined by the disk diffusion method, against the five fungal strains of medical importance, for the hybrid derivatives **5a**–**d** and **6a**–**d**. The positive control was fluconazole and nystatin while the negative control consists of sterile filter paper disks (with no antimicrobial compounds) soaked with DMSO 3%. The results of the antifungal assay are displayed as diameters of inhibition zones (in mm), where the larger diameter is the most active compound.

The data from Table 2 reveal that some of the hybrid *bis*-(imidazole/benzimidazole)-pyridine derivatives **5a**–**d** and **6a**–**d** manifest a good antifungal activity against the tested strains, the hybrid derivatives **5a** and **6a** have a quasi-nonselective antifungal activity, while the hybrids **5c** and **6c** are active against *C. albicans* wild type only. Against the fungal strains *C. parapsilosis* ATCC 22019, *C. parapsilosis* wild type and *Rhodotorula* sp., the *bis*-(imidazole)-pyridine hybrid **5a** (Y = -Ph) have excellent antifungal activity (with diameters of inhibition zones of 20 mm, 19 mm, 19 mm), superior or equal to the control drug *fluconazole* (18 mm, 19 mm, 19 mm). Against the remaining two strains (*C. albicans* wild type and *C. albicans* ATCC 10231) the hybrid **5a** has good-to-moderate antifungal activity (18 mm and 23 mm), close to the control drug *fluconazole* (26 mm and 24 mm). The *bis*-(benzimidazole)-pyridine hybrid **6a** (Y = -Ph) also has good antifungal activity against all five fungal strains (diameters of inhibition zones in the range of 7.5–13 mm), inferior to control drug *fluconazole* but still significant.

The *bis*-(imidazole)-pyridine hybrid **5c** (Y = -Br) and *bis*-(benzimidazole)-pyridine hybrid **6c** (Y = -Br) manifest a weak antifungal activity only against *C. albicans* wild type, with a diameter of inhibition zones of 7 mm and 7.5 mm, respectively.

In the next step of the antifungal assay, for the four active hybrid derivatives **5a**, **5c**, **6a** and **6c**, the minimum inhibitory concentration (MIC) was determined using the broth microdilution assay method [46,47]. The results of the MIC antifungal assay are displayed in Table 3.

The data from Table 3 reveal that the tested hybrid derivatives are active to a low concentration against the tested fungal strains, with a MIC value in the range of 3.9 to 62.5 µg/mL. The *bis*-(imidazole/benzimidazole)-pyridine derivatives **5a** and **6a** (with phenyl group (Y = -Ph) at the *para* position of the benzoyl moiety) are the most active (MIC in the range of 3.9 to 31.25 µg/mL), the best antifungal activity being against the fungal strains *C. albicans* wild type (MIC = 3.9 µg/mL in the case of hybrid **5a**) and *Rhodotorula* sp. (MIC = 3.9 µg/mL in the case of hybrid **6a**). The *bis*-(imidazole)-pyridine hybrid **5c** (Y = -Br) and *bis*-(benzimidazole)-pyridine hybrid **6c** (Y = -Br) manifest significant antifungal activity only against *C. albicans* wild type, with a MIC value of 62.5 µg/mL.

The *in vitro* antifungal assay against the four phytopathogenic filamentous fungal strains reveal that, from the tested hybrid *bis*-(imidazole/benzimidazole)-pyridine derivatives **5a**–**d** and **6a**–**d,** only one compound, namely the *bis*-(imidazole)-pyridine hybrid **5a** (Y = -Ph), manifests a strong selective antifungal activity against *A. niger* and *A. flavus*, Table 4.

Thus, the hybrid **5a** have a diameter of inhibition zone of 12 mm against *A. niger* and 9 mm against *A. flavus*, close to the control drug fluconazole (9 mm and 0 mm). The MIC assay (Table 5) for the hybrid **5a** indicates a value of 62.5 µg/mL against *A. niger* and 31.25 µg/mL against *A. flavus*, compared to the control drug nystatin (MIC of 0.48 µg/mL against both *A. niger* and *A. flavus*).

We may notice that the MIC values for **5a** against the fungal strain *A. flavus* are clearly higher than the MIC for nystatin; however, the values obtained are smaller than MIC obtained for many antifungal agents commercially used for plant protection [48,49].

From the SAR point of view, the comparative analysis of the data presented above denotes that the presence of a hybrid *bis*-(imidazole/benzimidazole)-pyridine moiety has a beneficial influence for antifungal activity against both human and plant pathogenic fungal strains. The substituent Y from the *para*/(4)-position of the benzoyl moiety anchored onto a *N*^3^-imidazole nitrogen atom plays also a crucial role in increasing the antifungal properties, with the presence of a phenyl ring (Y = -C_6_H_5_) being extremely beneficial for this activity; the presence of a bromine halogen moiety (Y = -Br) is also favorable to increase antifungal activity.

## 3. Materials and Methods

### 3.1. Materials and Measurements

2,6-*bis*(chloromethyl)pyridine (99% purity), imidazole (ACS reagent, ≥99% (titration)), benzimidazole (purity: >98.0%(GC)(T)), *p*-R (-Ph, -Cl, -Br, -NO_2_)-bromoacetophenones (99% purity) and sodium hydride (60% in mineral oil) were purchased from TCI and Sigma-Aldrich (Burlington, MA, USA). The NMR spectra were recorded on a Bruker Avance III 500 MHz spectrometer (Bruker, Vienna, Austria) operating at 500 MHz for ^1^H and 125 MHz for ^13^C, equipped with a 5 mm PABBO detection probe. The program used for acquisition and processing of data was TopSpin 3.2 PL5 (Bruker, Vienna, Austria). The abbreviations utilized to designate chemical shift multiplicities are: s = singlet, d = doublet, dd = doublet of doublets, add = apparent doublet of doublets, t = triplet, m = multiplet. Ultrasound-assisted reactions were performed using Sonics VCX-130, USA, with a nominal power of 130 W and a frequency of 20 kHz. For this ultrasonic reactor the titanium horn (diameter: 6 mm; length: 116 mm) was fixed to the ultrasonic converter. The titanium probe tip was directly immersed in the reaction mixture. Infrared (IR) data were recorded with FTIR Cary 630 spectrophotometer (Agilent Technologies, Mulgrave, Australia) coupled to a ZnSe ATR module for measuring solid samples. Mel-Temp apparatus (Barnstead International, Dubuque, IA, USA)was used to measure the melting points of compounds and are uncorrected. Thin layer chromatography (TLC) was performed on Merck silica gel 60 F_254_ plates (Merck, Darmstadt, Germany). The ultrasonic bath Elma Transsonic T310 (ELMA, Stuttgart, Germany) (power 34.5 W, frequency 35 kHz) was used for solubilizing the starting materials. Elemental analyses were done using a FlashSmart CHNS/O Elemental Analyzer (Thermo Fisher, Waltham, MA, USA), with MVC. The microanalyses were in satisfactory agreement with the calculated values: C, ±0.15; H, ±0.10; N, ±0.30.

### 3.2. Antifungal Assay

Tested compounds have been dissolved in 20% DMSO to a final concentration of 1 mg mL^−1^. All the reagents used were purchased from Merck (Darmstadt, Germany).

#### 3.2.1. Antifungal Effect Assessment

Evaluation of susceptibility to the new synthesized compounds have been determined using the Kirby–Bauer disk diffusion method, on 9 cm diameter Petri dishes with Sabouraud-agar, inoculated with suspensions of 10^6^ CFU/mL. Tested compounds were used at 1 mg/mL final concentration per each 6 mm diameter disk. Negative controls (20% DMSO) and positive controls (nystatin 200 μg/mL and fluconazole 32 μg/mL) were also used. The cultures were incubated at 37 °C for 48 h in the case of yeasts, and at 28 °C for 5 days in the case of filamentous fungi. All experiments have been done in triplicate and repeated. The results were calculated as an average of three replicates and three diameters measured for each disk.

#### 3.2.2. Determination of Minimum Inhibitory Concentration

Determination of minimum inhibitory concentration has been achieved only for the compounds that manifested inhibition of fungal growth in previous tests. The technique used was the broth microdilution method (MIC), following recommendation from the Clinical Standard Laboratory Institute, on 96-well round bottom plates for yeasts (with a final volume of 200 μL mixture per well) and 48-well plates for filamentous fungi (with a final volume of 400 μL mixture per well), in Sabouraud broth. Serial dilutions started at 250 μg/mL (5% DMSO) in the first well, being reduced to half subsequently. Controls with nystatin (200 μg mL^−1^ initial concentration) and fluconazole (32 μg/mL initial concentration) as standard antimycotics were used. Sterility controls (un-inoculated well containing media) and negative controls (inoculated wells with simple media) were also used. Inoculation has been performed with suspension of CFU as described, at a final ratio of 1:1 (inoculum versus supplemented media). Incubation was done at 37 °C for 48 h for yeasts, and at 28 °C for 3 days for filamentous fungi. A solution of 0.05% resazurin was used to read the results after incubation.

The synthesis and spectral characterization of starting materials (**3a** and **3b**) and the synthesis under conventional TH of compounds **5b**–**d** and **6b**–**d** (see also Supplementary Materials), were described in a previous work published by our group [40]. The complete spectral characterization of the hybrid molecules **5b**–**d** and **6b**–**d** can be found there.

### 3.3. General Procedure for the Synthesis of Quaternary Salts ***5a***–***d*** and ***6a***–***d*** Under US Irradiation

The 2,6-*bis*(imidazolium/benzimidazolium) salts were obtained according to the following method: intermediate **3a** or **3b** (1 mmol) is solubilized in approximately 40 mL of acetone and *p*-*R* (-Ph, -Cl, -Br, -NO_2_)-bromoacetophenone (**4a**–**d**) (2.4 mmol → **3a** and 4.5 mmol → **3b**) is gradually added after solubilization in approximately 20 mL of acetone. The reaction mixture was then ultrasonicated for approximately 100 min (parameters for sonication: A = 100%, 5 s ON/5 s OFF pulses). TLC was used to follow the progress of the reaction. The formed precipitates were collected by filtration, washed with acetone (3 times with 10 mL) and dried in vacuum. No other purification was required.

### 3.4. Spectral Data of Quaternary Salts ***5a***–***d*** and ***6a***–***d*** (Figure 3)

In Figure 3 is presented the numbering of atoms from compounds **5a**–**d** and **6a**–**d**, which is also used in the description of spectral data of the hybrid derivatives.

1,1′-(pyridine-2,6-diyl*bis*(methylene))*bis*(3-(2-([1,1′-biphenyl]-4-yl)-2-oxoethyl)-1*H*-imidazol-3-ium) (**5a**)

White powder; TH: η = 56%, US: η = 61%; m.p. = 226–229 °C. **^1^H-NMR** (500 MHz, DMSO-*d*_6_): δ = 5.73 (s, 4H, 2 × CH_2_-_α_,), 6.16 (s, 4H, 2 × CH_2_-_β_), 7.51–7.45 (m, 6H, 4 × H_8″_, 2 × H_9″_), 7.59–7.57 (d, *J* = 7.5 Hz, 2H, 2 × H_3_), 7.74–7.73 (d, *J* = 8.5 Hz, 4H, 4 × H_7″_), 7.80–7.79 (add, 4H, 2 × H_4′_, 2 × H_5′_), 7.90–7.88 (d, *J* = 8.5 Hz, 4H, 4 × H_4″_), 8.06–8.03 (t, *J* = 8.0 Hz, 1H, H_4_), 8.11–8.09 (d, *J* = 8.5 Hz, 4H, 4 × H_3″_), 9.24 (s, 2H, 2 × H_2′_). **^13^C-NMR** (125 MHz, DMSO-*d*_6_): δ = 52.7, 55.6, 122.2, 123.0, 123.9, 127.0, 127.0, 128.7, 128.9, 129.1, 132.3, 137.9, 138.3, 138.9, 145.7, 153.6, 191.0. **IR** (ATR, ν(cm^−1^)): 3017, 2986, 1695, 1583, 1251. Anal. Calcd. for C_41_H_35_Br_2_N_5_O_2_: C, 62.37; H, 4.47; N, 8.87; Found: C, 62.47; H, 4.37; N, 8.67.

1,1′-(pyridine-2,6-diyl*bis*(methylene))*bis*(3-(2-([1,1′-biphenyl]-4-yl)-2-oxoethyl)-1*H*-benzo[d]imidazol-3-ium) (**6a**)

White powder; TH: η = 46%, US: η = 64%; m.p. = 213–215 °C. **^1^H-NMR** (500 MHz, DMSO-*d*_6_): 6.03 (s, 4H, 2 × CH_2_-_α_), 6.50 (s, 4H, 2 × CH_2_-_β_), 7.52–7.44 (m, 8H, 2 × H_6′,_ 2 × H_9″,_ 4 × H_8″_), 7.63–7.60 (t, *J* = 8.0 Hz, 2H, 2 × H_5′_), 7.71–7.69 (d, 2H, 2 × H_7′_, *J* = 8.0 Hz), 7.78–7.74 (m, 6H, 2 × H_3_, 4 × H_7″_), 7.92–7.91 (d, *J* = 8.5 Hz, 4H, 4 × H_4″_), 8.09–8.08 (m, 3H, 2 × H_4′_, H_4_), 8.18–8.16 (d, 4H, 4 × H_3″_), 9.90 (s, 2H, 2 × H_2′_). **^13^C-NMR** (125 MHz, DMSO-*d*_6_): 50.6, 53.41, 113.48, 113.98, 122.81, 126.57, 126.74, 127.07, 128.76, 129.28, 131.65, 132.72, 133.4, 138.46, 139.06, 143.87, 145.81, 153.09, 190.91. **IR** (ATR, ν(cm^−1^)): 3020, 2973, 1673, 1591, 1545, 1467. Anal. Calcd. for C_49_H_39_Br_2_N_5_O_2_: C, 66.15; H, 4.42; N, 7.87; Found: C, 66.25; H, 4.32; N, 7.67.

## 4. Conclusions

In conclusion, we report herein the design, synthesis, structure and antifungal activity of a new class of hybrid *bis*-(imidazole/benzimidazole)-pyridine derivatives against human and plant pathogenic fungi. The method of synthesis for the hybrid salts is direct and efficient involving two subsequent *N*-alkylation reactions of the nitrogen atoms from an imidazole ring. The synthesis of the hybrid derivatives was carried out both under conventional thermal heating and ultrasound irradiation. Under ultrasound irradiation the reaction pathway has some certain advantages in terms of a dramatical decrease in reaction time (by four to seven folds), a remarkable decrease in the consumed energy, and higher yields (with about 10–15%). The structure of the hybrid *bis*-(imidazole/benzimidazole)-pyridine derivatives was determined by elemental and spectral (IR, NMR) analysis. The *in vitro* antifungal activity of the hybrid *bis*-(imidazole/benzimidazole)-pyridine derivatives was evaluated against human and plant pathogenic fungi and revealed that four hybrids [**5a** and **6a** (Y = -Ph), **5c** and **6c** (Y = -Br)], are active to a low concentration against the tested fungal strains, with a MIC value in the range of 3.9 to 62.5 µg/mL. The best antifungal activity was manifested by the hybrid *bis*-(imidazole)-pyridine **5a**, against both types of human and plant fungal strains, in some cases closely to the control drug fluconazole, which is superior to many antifungal agents commercially used for plant protection. The SAR correlations reveal that the presence of a hybrid *bis*-(imidazole/benzimidazole)-pyridine moiety has a beneficial influence on antifungal activity against both human and plant pathogenic fungal strains, and the presence of a phenyl ring (Y = -C_6_H_5_) at the *para* position of a benzoyl moiety from the imidazole/benzimidazole heterocycle, substantially increases antifungal properties. The results obtained make us conclude that the hybrid compound *bis*-(imidazole)-pyridine **5a**, could be considered as a good lead candidate for future drug or pesticide development, and future research in this respect will be done.

## Figures and Tables

**Table 1:** Comparative study regarding the synthesis of hybrid *bis*-(imidazolium/benzimidazolium) salts under TH (thermal heating) versus US (ultrasound irradiation).

	Compound	5a	5b	5c	5d	6a	6b	6c	6d
Yield (%) *	**TH**	56	87	84	79	46	71	55	48
	**US**	61	90	86	85	64	73	57	56
R.t. (min) **	**TH**	720	480	600	720	720	480	480	720
	**US**	120	100	100	100	120	120	120	120

* Yields were calculated in isolated product after purification; ** R.t = Reaction time.

**Table 2:** Antifungal assessment through Kirby–Bauer diffusion assay for fungal strains of medical importance.

Compound/Strain	Average Diameter of Inhibition Zone (mm) *^,^**				
	*CAW*	*CA10231*	*CPW*	*CP22019*	*RH*
**5a**	**18** ± 1.4	**23** ± 1.9	**19** ± 1.82	**20** ± 1.95	**18.5** ± 1.8
**5b**	0	0	0	0	0
**5c**	7 ± 0.1	0	0	0	0
**5d**	0	0	0	0	0
**6a**	**10** ± 0.8	7.5 ± 0.1	**8.5** ± 0.8	**10** ± 1.0	**13** ± 1.1
**6b**	0	0	0	0	0
**6c**	7.5 ± 0.03	0	0	0	0
**6d**	0	0	0	0	0
**Std-Flu**	**26** ± 2.15	**24.5** ± 2.1	**19** ± 1.85	**18** ± 1.82	**19** ± 1.75
**Std-Nyst**	**28** ± 2.18	**26** ± 2.14	**25.5** ± 2.05	**26.5** ± 1.95	**30** ± 2.6
***DMSO***	*0*	*0*	*0*	*0*	*0*

(*CAW*—*Candida albicans* wild type; *CA10231*—*C. albicans* ATCC 10231; *CPW*—*C. parapsilosis* wild type; *CP22019*—*C. parapsilosis* ATCC 22019; *RH*—*Rhodotorula* sp.; Std-Flu—reference antimycotic fluconazole; Std-Nyst—reference antimycotic nystatin; ±—standard deviation); * in bold are marked higher values of inhibition zone diameters; ** the values include the 6 mm diameter of the disks.

**Table 3:** Minimum inhibitory concentration (MIC) assessment for selected compounds against yeasts.

Compound/Strain	MIC (µg/mL)				
	*CAW*	*CA10231*	*CPW*	*CP2019*	*RH*
**5a**	3.9	15.62	31.25	31.25	15.62
**5c**	62.5	N	N	N	N
**6a**	15.62	31.25	31.25	31.25	3.9
**6c**	62.5	N	N	N	N
**Std-Flu**	0.12	0.12	0.97	7.81	0.48
**Std-Nyst**	0.24	0.24	0.97	0.24	0.12
**DMSO**	>250	>250	250	>250	125

(*CAW*—*Candida albicans* wild type; *CA10231*—*C. albicans* ATCC 10231; *CPW*—*C. parapsilosis* wild type; *CP22019*—*C. parapsilosis* ATCC 22019; *RH*—*Rhodotorula* sp.; Std-Flu—reference antimycotic fluconazole; Std-Nyst—reference antimycotic nystatin; N—not tested).

**Table 4:** Antifungal assessment through Kirby–Bauer diffusion assay.

Compound/Strain	Average Diameter of Inhibition Zone (mm) *^,^**			
	*AN*	*AF*	*CC*	*RN*
**5a**	**12** ± 1.0	9 ± 0.07	**9** ± 0.22	8 ± 0.03
**5b**	0	0	**16** ± 1.55	0
**5c**	0	0	8 ± 0.05	0
**5d**	0	0	0	0
**6a**	0	0	8 ± 0.05	7 ± 0.1
**6b**	0	0	0	0
**6c**	0	0	0	0
**6d**	0	0	0	0
**Std-Flu**	**9** ± 0.9	0	**24** ± 2.1	0
**Std-Nyst**	**20.5** ± 1.85	**13.5** ± 1.24	**11** ± 1.05	0
**DMSO**	0	0	0	0

*AN*—*Aspergillus niger*; *AF*—*A. flavus*; *CC*—*Cladosporium cladosporioides*; *RN*—*Rhizopus nigricans*; Std-Flu—reference antimycotic fluconazole; Std-Nyst—reference antimycotic nystatin; ±—standard deviation); * in bold are marked higher values of inhibition zone diameters; ** the values include the 6 mm diameter of the disks.

**Table 5:** Minimum inhibitory concentration assessment for selected compounds against filamentous fungi (Std-Nyst—nystatin, N-not tested).

Compound/Strain	MIC (µg/mL)			
	*AN*	*AF*	*CC*	*RN*
**5a**	62.5	31.25	250	250
**6a**	N	N	N	250
**Std-Nyst**	0.48	0.48	125	>250
**DMSO**	0	0	0	0

*AN*—*Aspergillus niger*; *AF*—*A. flavus*; *CC*—*Cladosporium cladosporioides*; *RN*—*Rhizopus nigricans*; Std-Nyst—nystatin; N—not tested.

## Data Availability

The original contributions presented in the study are included in the article/Supplementary Materials, further inquiries can be directed to the corresponding authors.

## Supplementary Materials

The following supporting information can be downloaded at: https://www.mdpi.com/article/10.3390/ph18040495/s1, Figure S1: 1H-NMR Spectrum of compound 5a; Figure S2: 13C-NMR Spectrum of compound 5a; Figure S3. 1H-NMR Spectrum of compound 6a; Figure S4. 13C-NMR Spectrum of compound 6a; Figure S5. 1H-NMR Spectrum of compound 5b; Figure S6. 13C-NMR Spectrum of compound 5b; Figure S7. 1H-NMR Spectrum of compound 5c; Figure S8. 13C-NMR Spectrum of compound 5c; Figure S9. 1H-NMR Spectrum of compound 5d; Figure S10. 13C-NMR Spectrum of compound 5d; Figure S11. 1H-NMR Spectrum of compound 6b; Figure S12. 13C-NMR Spectrum of compound 6b; Figure S13. 1H-NMR Spectrum of compound 6c; Figure S14. 13C-NMR Spectrum of compound 6c; Figure S15. 1H-NMR Spectrum of compound 6d; Figure S16. 13C-NMR Spectrum of compound 6d.
