# Supplementary material for: Hybrid Bis-(Imidazole/Benzimidazole)-Pyridine Derivatives with Antifungal Activity of Potential Interest in Medicine and Agriculture via Improved Efficiency Methods
Source: Pharmaceuticals (Basel). 2025 Mar 28;18(4):495. doi: 10.3390/ph18040495 (PMC12030448; doi:10.3390/ph18040495)
Supplement: Supplementary file 1 [file pharmaceuticals-18-00495-s001.zip › pharmaceuticals-3521302-supplementary.pdf]

pharmaceuticals

Article

Hybrid *Bis*-(Imidazole/Benzimidazole)-Pyridine Derivatives with Antifungal Activity of Potential Interest in Medicine and Agriculture via Improved Efficiency Methods

Tiberius Balaes [1], Violeta Mangalagiu [2,3], Vasilichia Antoci [4], Dorina Amariucai-Mantu [4], Dumitrela Diaconu [4,5,*] and Ionel I. Mangalagiu [4,*]

[1] Department of Biology, Faculty of Biology, Alexandru Ioan Cuza University of Iasi, 20A Carol 1st Bvd, Iasi 700505, Romania; tiberius.balaes@gmail.cm
[2] Institute of Interdisciplinary Research, CERNESIM Centre, Alexandru Ioan Cuza University of Iasi, Bd. Carol I, No. 11, 700506 Iasi, Romania; violeta.mangalagiu@uaic.ro
[3] Faculty of Food Engineering, Stefan Cel Mare University of Suceava, 13 Universitatii Str., 720229 Suceava, Romania
[4] Faculty of Chemistry, Alexandru Ioan Cuza University of Iasi, Bd. Carol I, No. 11, 700506 Iasi, Romania; vasilichia.antoci@uaic.ro (V.A.); dorina.mantu@uaic.ro (D.A.-M.)
[5] Institute of Interdisciplinary Research, RECENT-AIR Centre, Alexandru Ioan Cuza University of Iasi, Bd. Carol I, No. 11, 700506 Iasi, Romania
* Correspondence: dumitrela.diaconu@uaic.ro (D.D.); ionelm@uaic.ro (I.I.M.)

Content

Figure S1. ^{1}H-NMR Spectrum of compound **5a** 2
Figure S2. ^{13}C-NMR Spectrum of compound **5a** 2
Figure S3. ^{1}H-NMR Spectrum of compound **6a** 3
Figure S4. ^{13}C-NMR Spectrum of compound **6a** 3
Figure S5. ^{1}H-NMR Spectrum of compound **5b** 4
Figure S6. ^{13}C-NMR Spectrum of compound **5b** 4
Figure S7. ^{1}H-NMR Spectrum of compound **5c** 5
Figure S8. ^{13}C-NMR Spectrum of compound **5c** 5
Figure S9. ^{1}H-NMR Spectrum of compound **5d** 6
Figure S10. ^{13}C-NMR Spectrum of compound **5d** 6
Figure S11. ^{1}H-NMR Spectrum of compound **6b** 7
Figure S12. ^{13}C-NMR Spectrum of compound **6b** 7
Figure S13. ^{1}H-NMR Spectrum of compound **6c** 8
Figure S14. ^{13}C-NMR Spectrum of compound **6c** 8
Figure S15. ^{1}H-NMR Spectrum of compound **6d** 9
Figure S16. ^{13}C-NMR Spectrum of compound **6d** 9
NMR spectral data of compounds 5b-d and 6b-d 10-11
General procedure for synthesis of quaternary salts 5a-g and 6a-g under conventional TH 11

The NMR spectra were recorded on a Bruker Avance III 500 MHz spectrometer operating at 500 MHz for 1H and 125 MHz for 13C. The NMR apparatus is equipped with a 5 mm PABBO detection probe, and the program used for acquisition and processing of data was TopSpin 3.2 PL5.

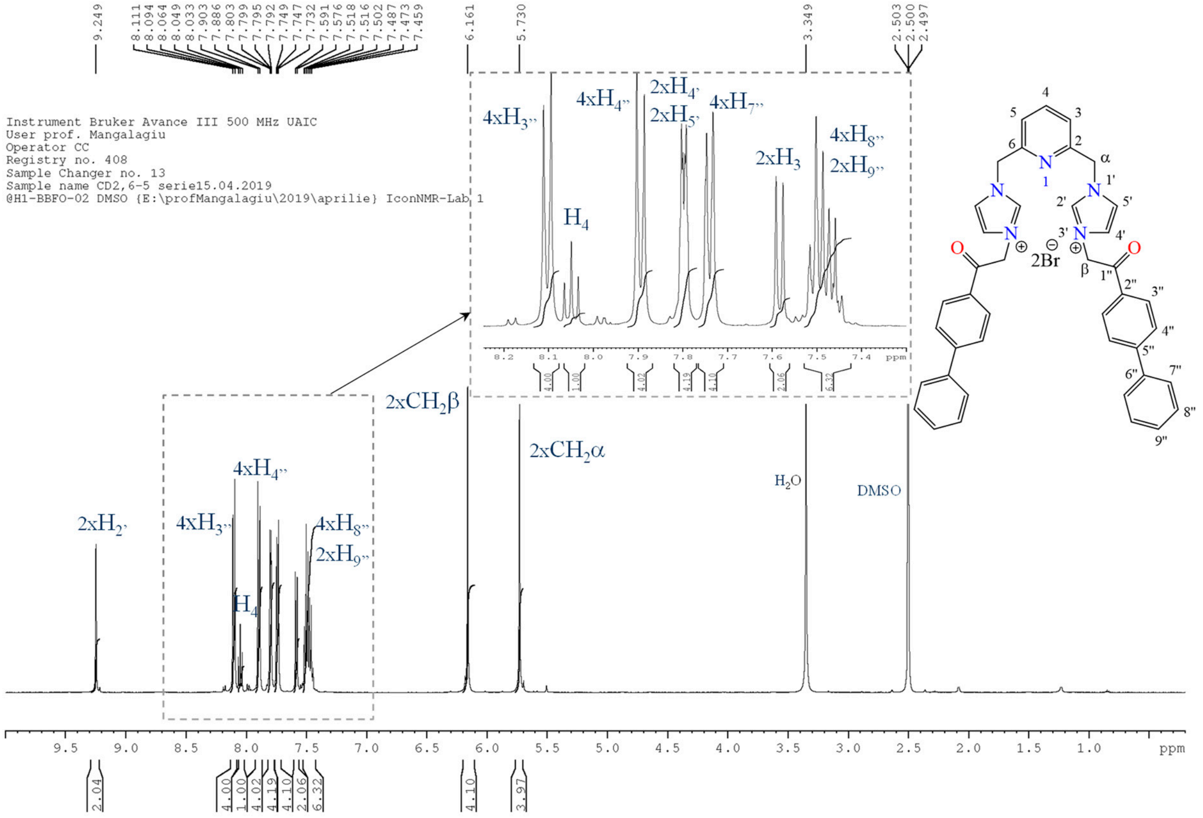

Figure S1: ^{1}H-NMR Spectrum of compound **5a**

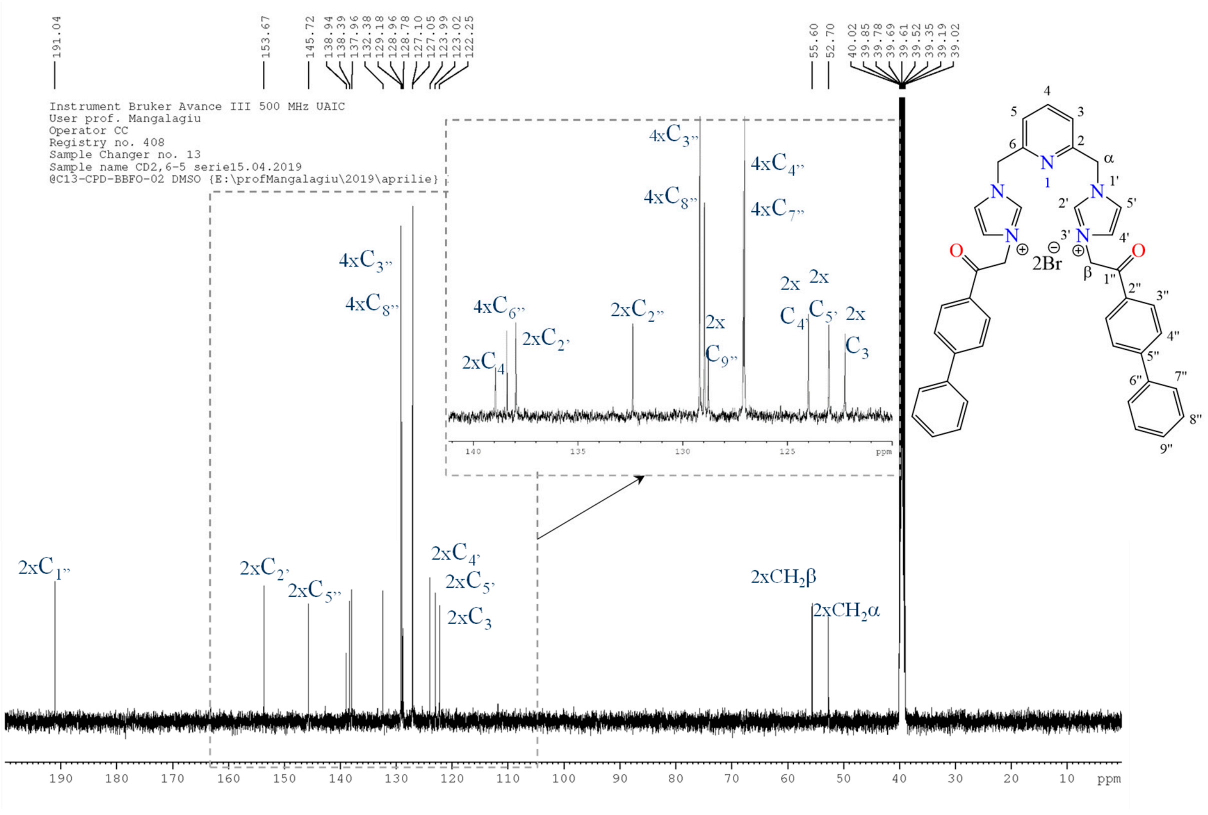

Figure S2: ^{13}C-NMR Spectrum of compound **5a**

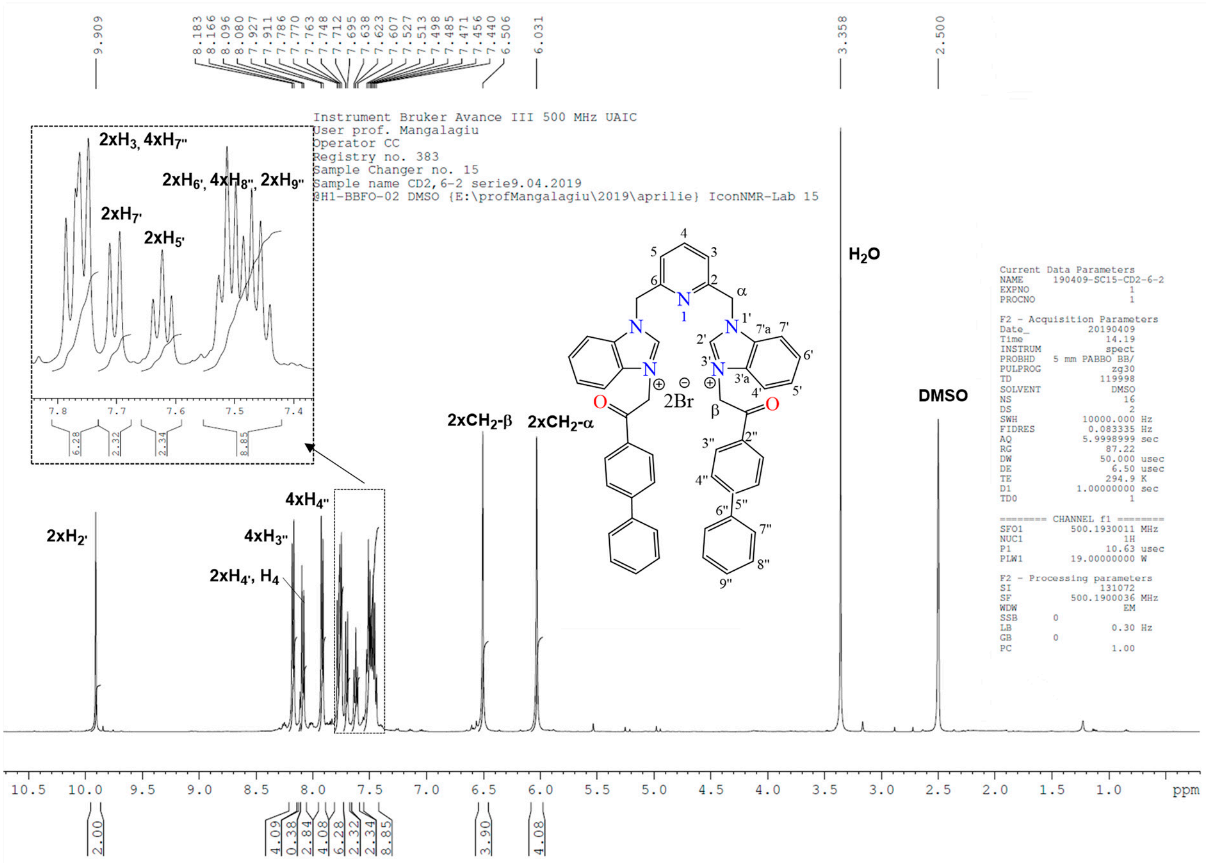

Figure S3. ^{1}H-NMR Spectrum of compound **6a**

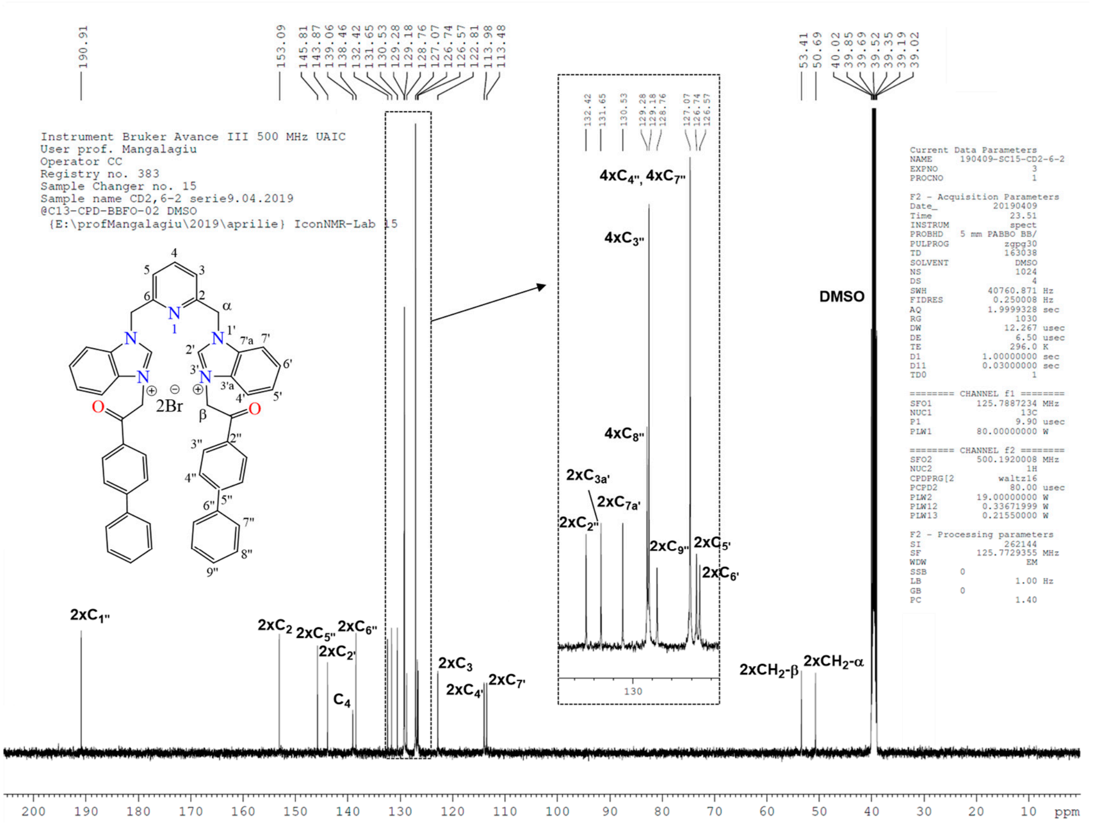

Figure S4. ^{13}C-NMR Spectrum of compound **6a**

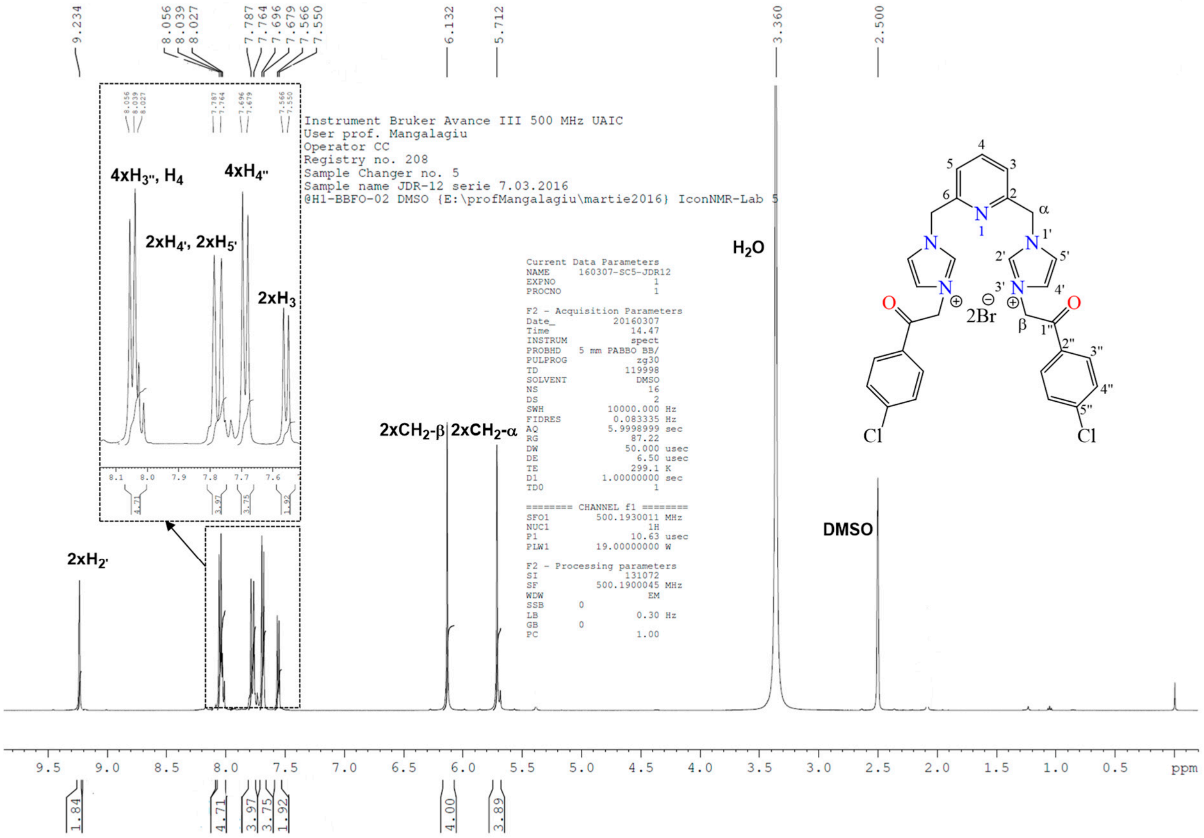

Figure S5. ^{1}H-NMR Spectrum of compound **5b**

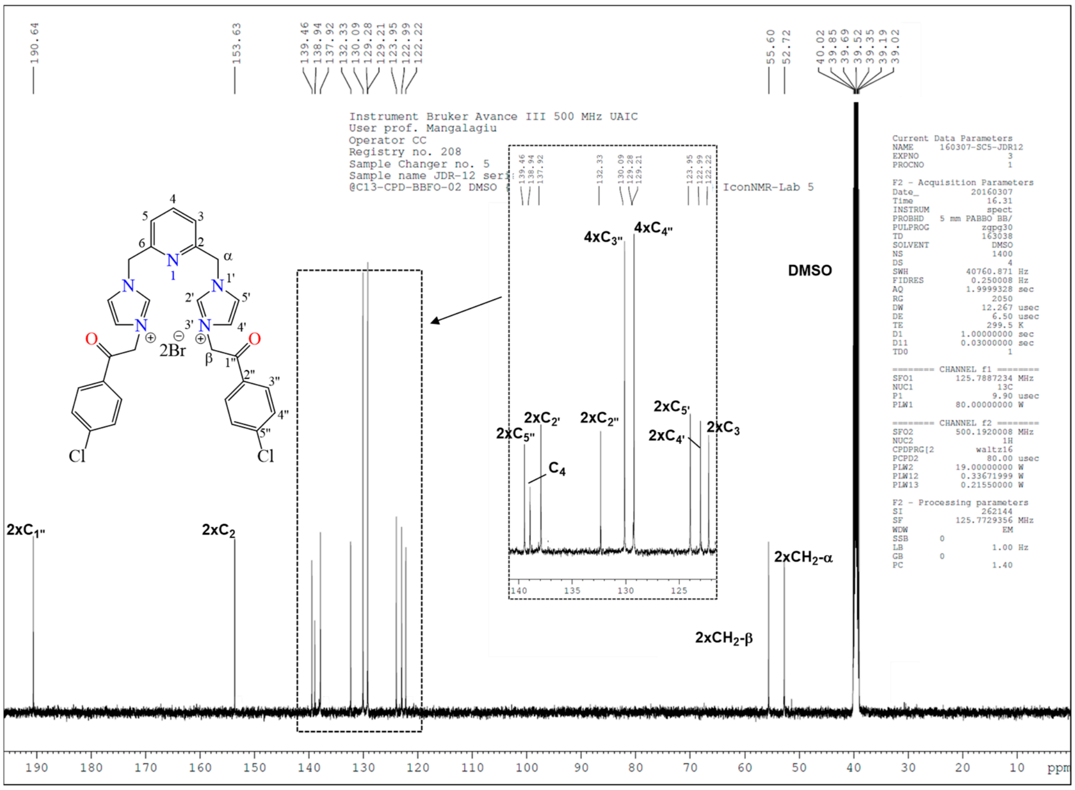

Figure S6. ^{13}C-NMR Spectrum of compound **5b**

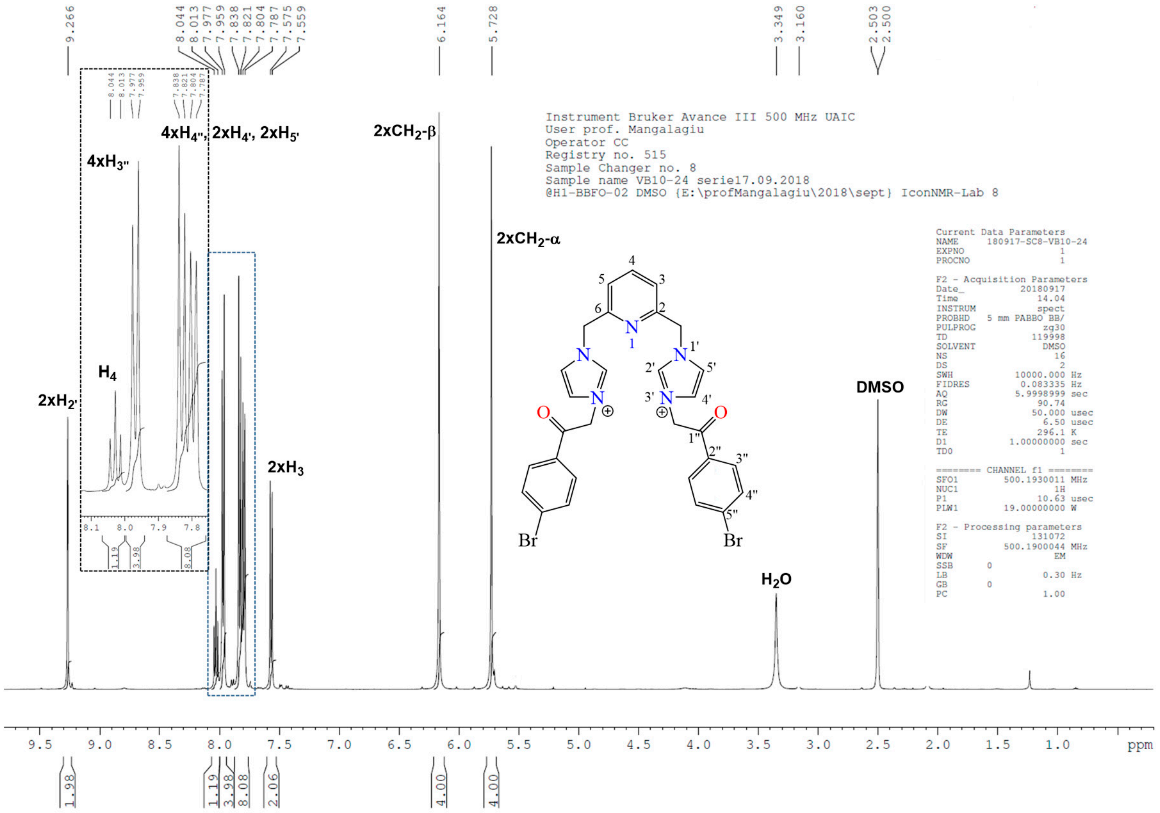

Figure S7. ^{1}H-NMR Spectrum of compound **5c**

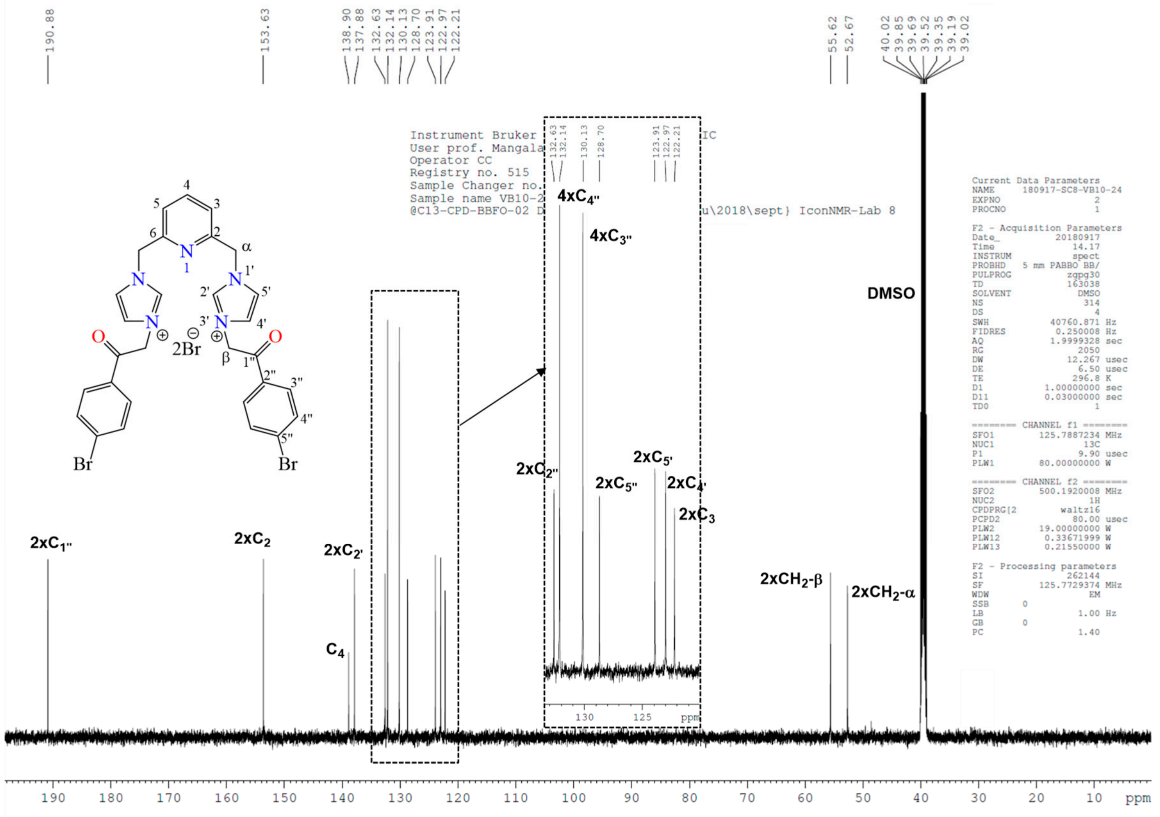

Figure S8. ^{13}C-NMR Spectrum of compound **5c**

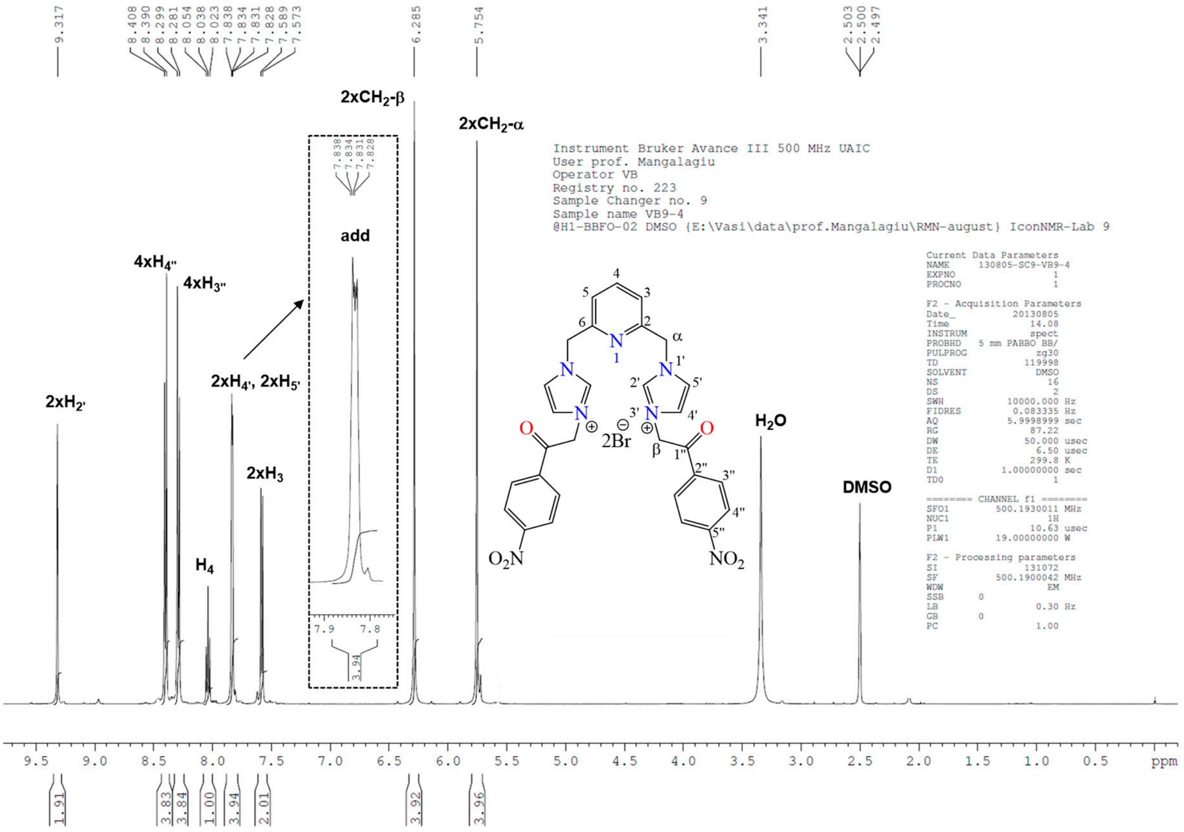

Figure S9. ^{1}H-NMR Spectrum of compound **5d**

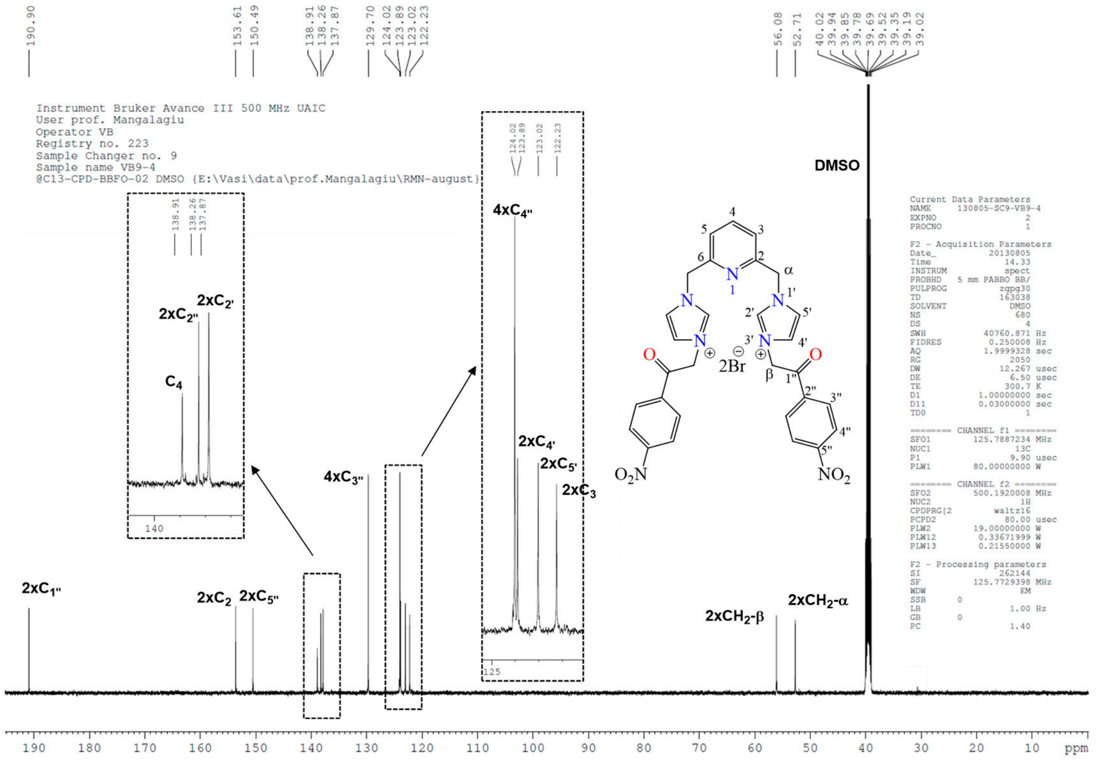

Figure S10. ^{13}C-NMR Spectrum of compound **5d**

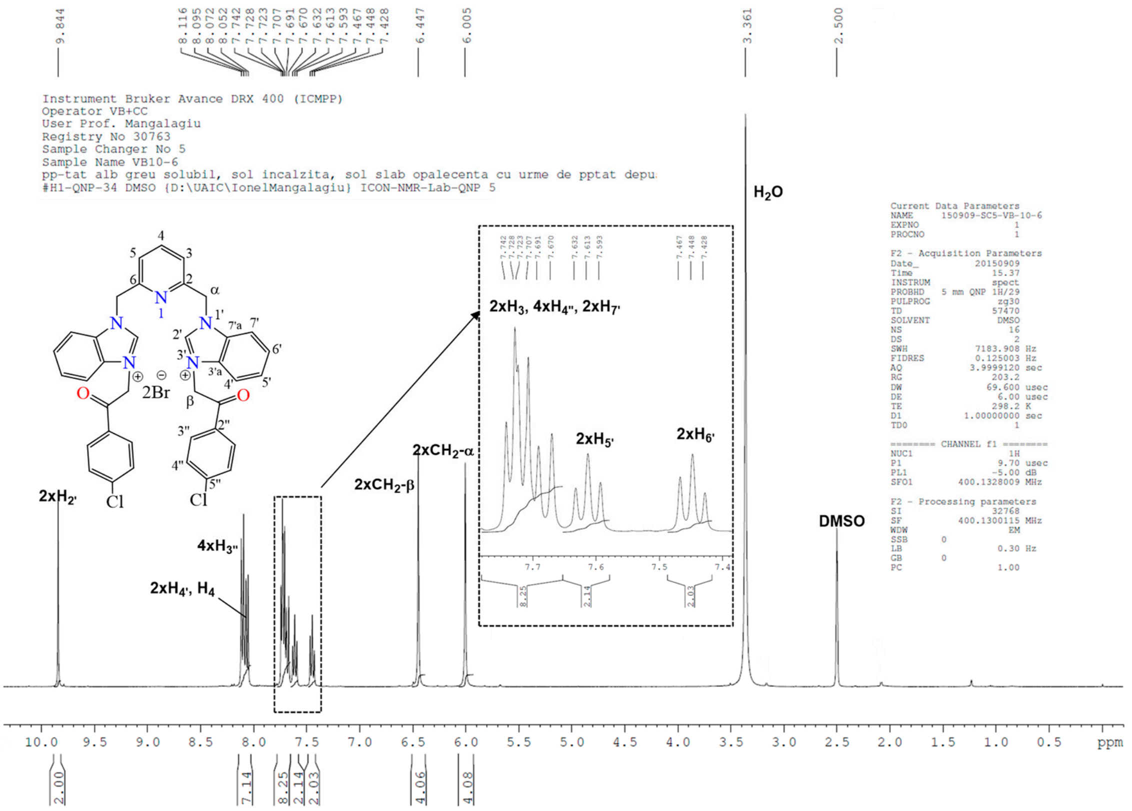

Figure S11. ^{1}H-NMR Spectrum of compound **6b**

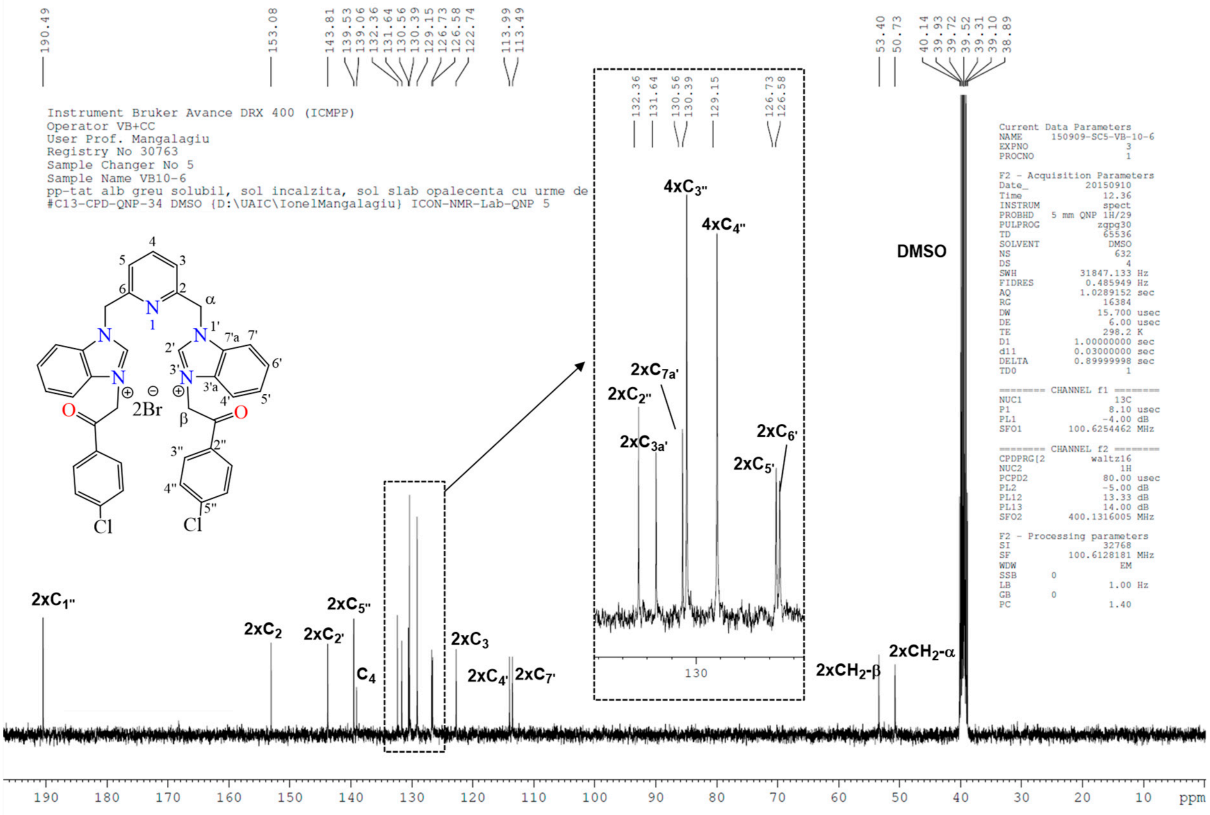

Figure S12. ^{13}C-NMR Spectrum of compound **6b**

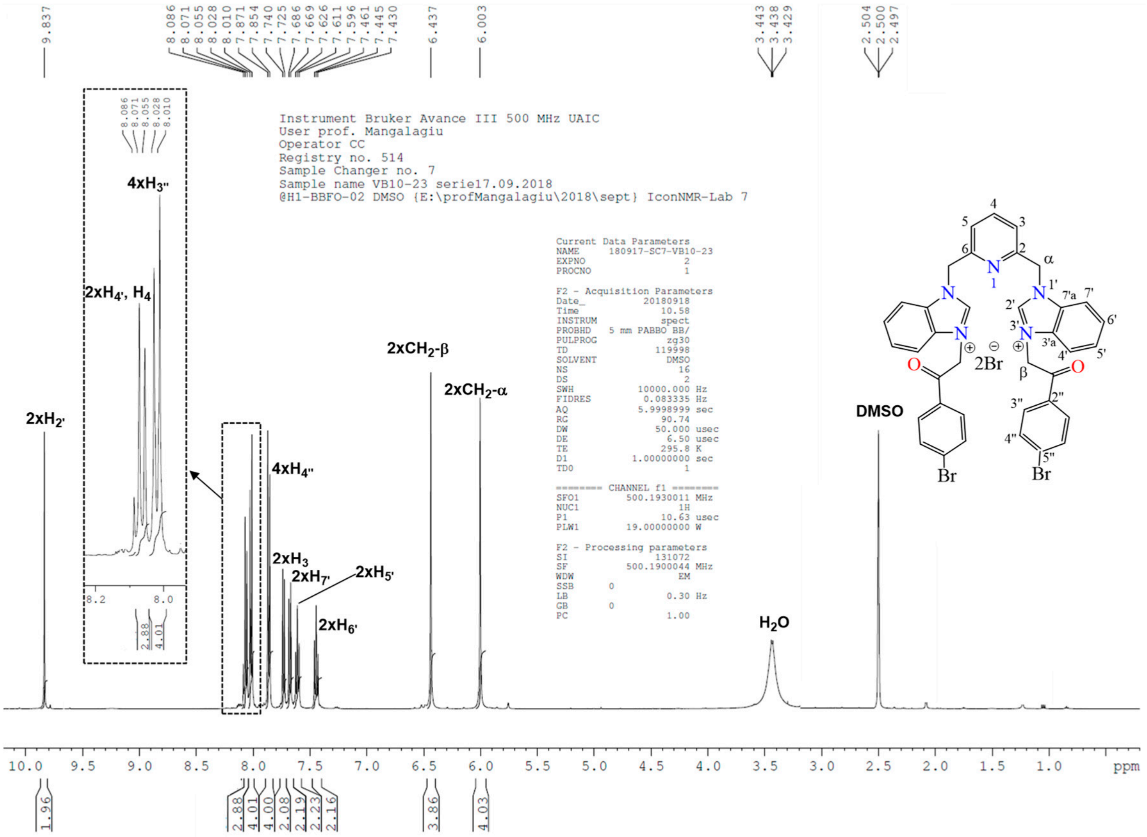

Figure S13. ^{1}H-NMR Spectrum of compound **6c**

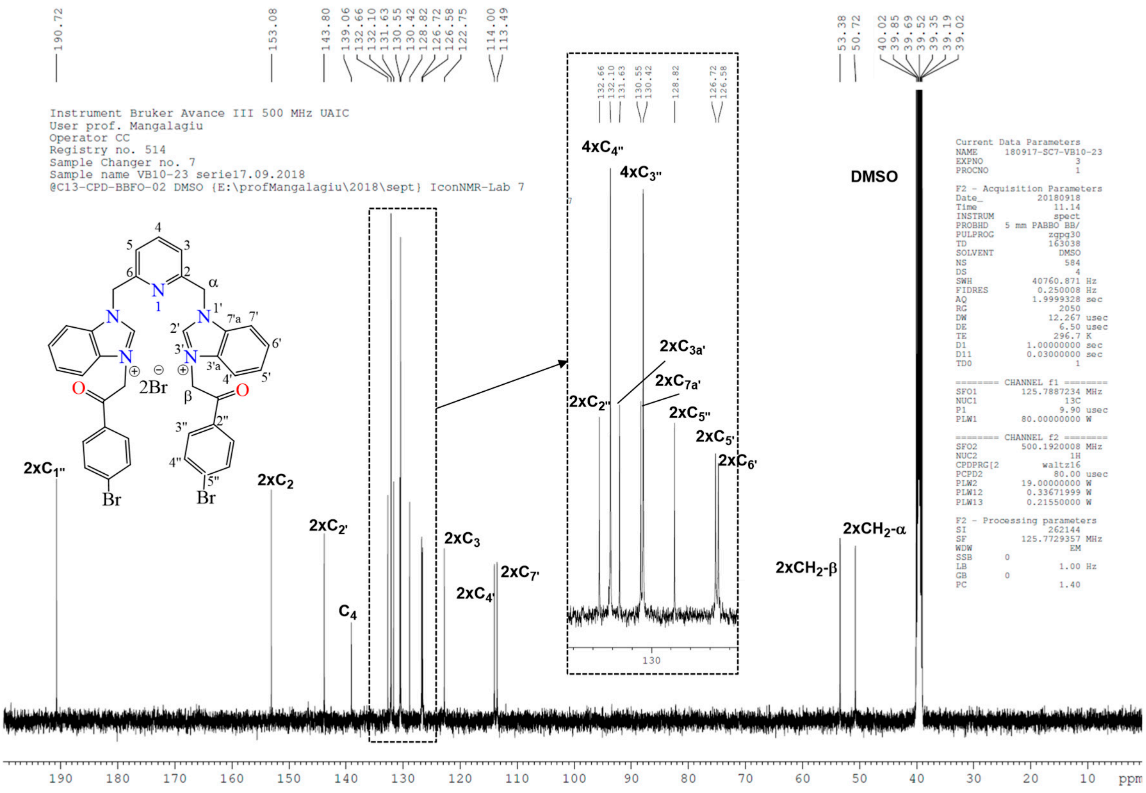

Figure S14. ^{13}C-NMR Spectrum of compound **6c**

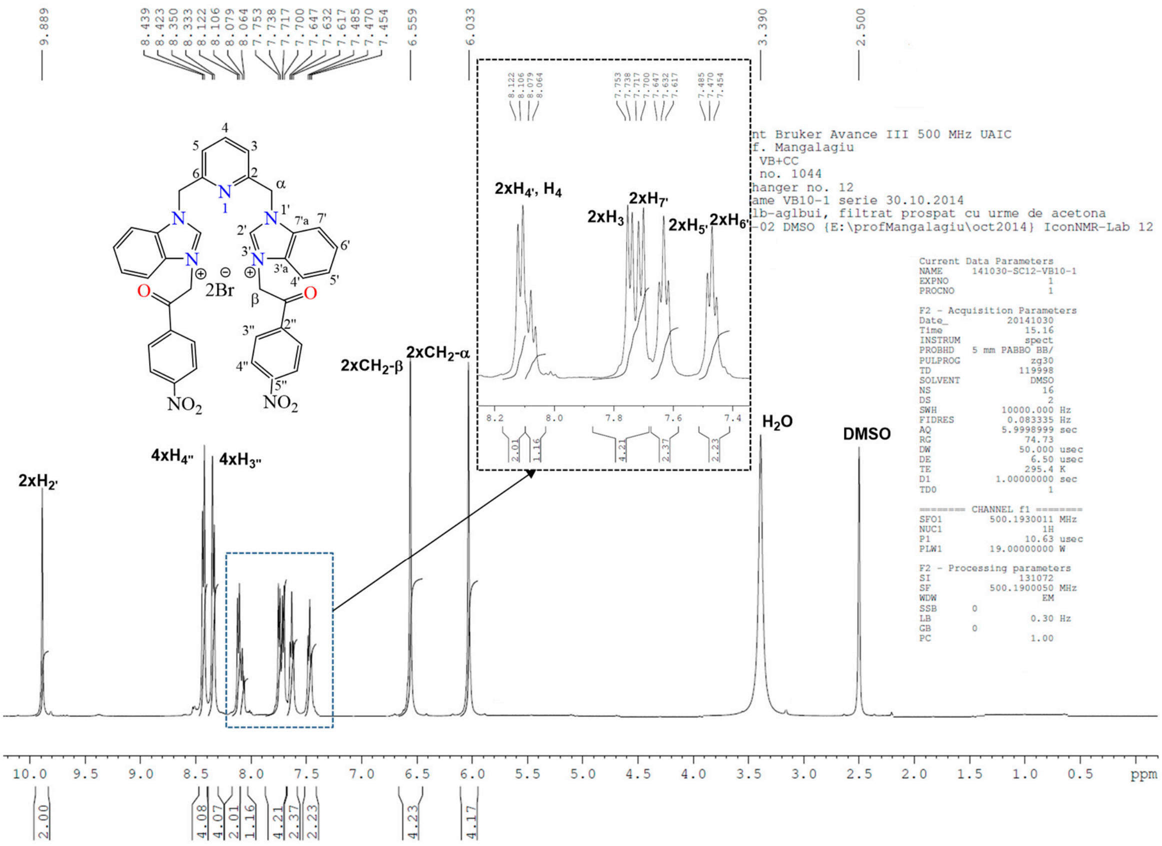

Figure S15. ^{1}H-NMR Spectrum of compound **6d**

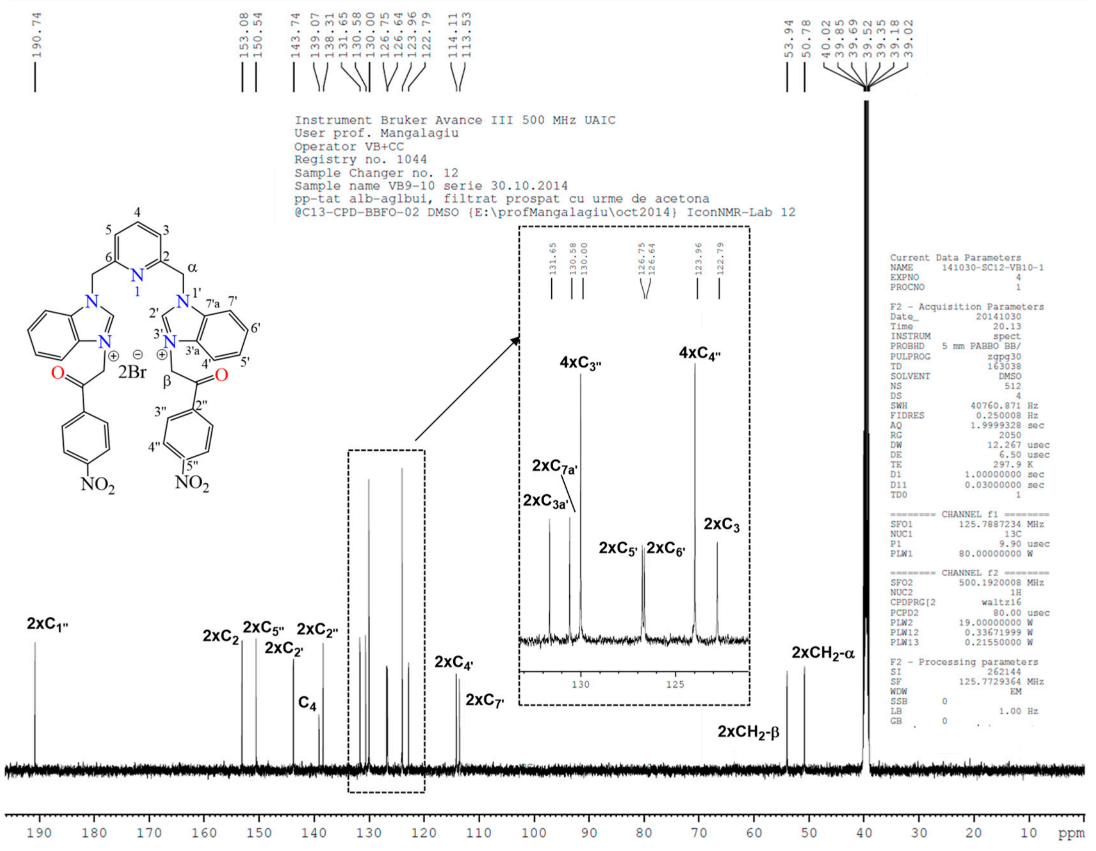

Figure S16. ^{13}C-NMR Spectrum of compound **6d**

NMR spectral data of compounds 5b-d and 6b-d

1,1′-(pyridine-2,6-diylbis(methylene))bis(3-(2-(4-chlorophenyl)-2-oxoethyl)-1H-imidazol-3-ium) dibromide **(5b)**

White powder; US: η= 90%; m.p.= 209-212 °C. ^{1}**H-NMR** (500 MHz, DMSO-*d*$_6$): δ= 5.71 (s, 4H, 2xCH$_{2}$-α), 6.13 (s, 4H, 2xCH$_{2}$-β), 7.56 (d, *J*= 8.0 Hz, 2H, 2xH$_3$), 7.69 (d, *J*= 8.5 Hz, 4H, 4xH$_{4''}$), 7.76 (s, 2H, 2xH$_{5'}$), 7.79 (s, 2H, 2xH$_{4'}$), 8.06-8.03 (m, 5H, 4xH$_{3''}$, H$_4$), 9.23 (s, 2H, 2xH$_{2'}$). ^{13}**C-NMR** (125 MHz, DMSO-*d*$_6$): δ= 52.7, 55.6, 122.2, 122.9, 123.9, 129.2, 130.1, 132.3, 137.9, 138.9, 139.5, 153.6, 190.6.

1,1′-(pyridine-2,6-diylbis(methylene))bis(3-(2-(4-bromophenyl)-2-oxoethyl)-1H-imidazol-3-ium) dibromide **(5c)**

White powder; US: η= 86%; m.p.= 238-240 °C. ^{1}**H-NMR** (500 MHz, DMSO-*d*$_6$): δ= 5.73 (s, 4H, 2xCH2-α), 6.16 (s, 4H, 2xCH2-β), 7.57 (d, *J*= 8.0 Hz, 2H, 2xH$_3$), 7.79 (s, 2H, 2xH$_{5'}$), 7.80 (s, 2H, 2xH$_{4'}$), 7.83 (d, *J*= 8.5 Hz, 4H, 4xH$_{4''}$), 7.97 (d, *J*= 8.5 Hz, 4H, 4xH$_{3''}$), 8.03 (t, *J*= 8.0 Hz, 1H, H$_4$), 9.27 (s, 2H, 2xH$_{2'}$). ^{13}**C-NMR** (125 MHz, DMSO-*d*$_6$): δ= 52.7, 55.6, 122.2, 122.9, 123.9, 128.7, 130.1, 132.1, 132.6, 137.9, 138.9, 153.6, 190.9.

1,1′-(pyridine-2,6-diylbis(methylene))bis(3-(2-(4-nitrophenyl)-2-oxoethyl)-1H-imidazol-3-ium) dibromide **(5d)**

Yellow powder; US: η= 85%; m.p.= 233-236 °C. ^{1}**H-NMR** (500 MHz, DMSO-*d*$_6$): δ= 5.75 (s, 4H, 2xCH$_{2}$-α), 6.28 (s, 4H, 2xCH$_{2}$-β), 7.58 (d, *J*= 8.0 Hz, 2H, 2xH$_3$), 7.83 (add, 4H, 2xH$_{4'}$, 2xH$_{5'}$), 8.04 (t, *J*= 8.0 Hz, 1H, H$_4$), 8.29 (d, *J*= 9.0 Hz, 4H, 4xH$_{3''}$), 8.40 (d, *J*= 9.0 Hz, 4H, 4xH$_{4''}$), 9.32 (s, 2H, 2xH$_{2'}$). ^{13}**C-NMR** (125 MHz, DMSO-*d*$_6$): δ= 52.7, 56.1, 122.2, 123.0, 123.9, 124.0,129.7, 137.9, 138.3, 138.9, 150.5, 153.6, 190.9.

(1,1′-(pyridine-2,6-diylbis(methylene))bis(3-(2-(4-chlorophenyl)-2-oxoethyl)-1H-benzo[d]imidazol-3-ium)) dibromide **(6b)**

White powder; η= 73%; m.p.= 175-178 °C. ^{1}**H-NMR** (500 MHz, DMSO-*d*$_6$): 6.00 (s, 4H, 2xCH$_{2}$-α), 6.45 (s, 4H, 2xCH$_{2}$-β), 7.45 (t, *J*= 7.6 Hz, 2H, 2xH$_{6'}$), 7.61 (t, *J*= 7.6 Hz, 2H, 2xH$_{5'}$), 7.74-7.67 (m, 8H, 2xH$_3$, 4xH$_{4''}$, 2xH$_{7'}$), 8.06 (d, *J*= 8 Hz, 3H, 2xH$_{4'}$, H$_4$), 8.10 (d, *J*= 8.4 Hz, 4H, 4xH$_{3''}$), 9.84 (s, 2H, 2xH$_{2'}$). ^{13}**C-NMR** (125 MHz, DMSO-*d*$_6$): 50.7, 53.4,113.5, 113.9, 122.7, 126.6, 126.7, 129.1, 130.4, 130.6, 131.6, 132.4,139.1, 139.5, 143.8, 153.1, 190.5.

(1,1′-(pyridine-2,6-diylbis(methylene))bis(3-(2-(4-bromophenyl)-2-oxoethyl)-1H-benzo[d]imidazol-3-ium)) dibromide **(6c)**

White powder; US: η= 57%; m.p.= 193-195 °C. ^{1}**H-NMR** (500 MHz, DMSO-*d*$_6$): 6.00 (s, 4H, 2xCH$_{2}$-α), 6.44 (s, 4H, 2xCH$_{2}$-β), 7.45 (t, *J*= 7.5 Hz, 2H, 2xH$_{6'}$), 7.61 (t, *J*= 7.5 Hz, 2H, 2xH$_{5'}$), 7.68 (d, *J*= 8.0 Hz, 2H, 2xH$_{7'}$), 7.73 (d, *J*= 7.5 Hz, 2H, 2xH$_{3,}$), 7.86 (d, *J*= 8.5 Hz, 4H, 4xH$_{4''}$), 8.02 (d, *J*= 8.5 Hz, 4H, 4xH$_{3''}$), 8.09-8.05 (m, 3H, H$_4$, 2xH$_{4'}$), 9.84 (s, 2H, 2xH$_{2'}$). ^{13}**C-NMR** (125 MHz, DMSO-*d*$_6$): 50.7, 53.4, 113.5, 114.0, 122.7, 126.6, 126.7, 128.8, 130.4, 130.5, 131.6, 132.1, 132.7, 139.1, 143.8, 153.1, 190.7.

(1,1′-(pyridine-2,6-diylbis(methylene))bis(3-(2-(4-nitrophenyl)-2-oxoethyl)-1H-benzo[d]imidazol-3-ium)) dibromide **(6d)**

Yellow powder; US: η= 56%; m.p.= 235-238 °C. **^{1}H-NMR** (500 MHz, DMSO-*d*$_6$): 6.03 (s, 4H, 2xCH$_2$-α), 6.56 (s, 4H, 2xCH$_2$-β), 7.47 (t, *J*= 7.5 Hz, 2H, 2xH$_{6'}$), 7.63 (t, *J*= 7.5 Hz, 2H, 2xH$_{5'}$), 7.71 (d, *J*= 8.5 Hz, 2H, 2xH$_{7'}$), 7.75 (d, *J*= 7.5 Hz, 2H, 2xH$_3$), 8.12-8.06 (m, 3H, 2xH$_{4'}$, H$_4$), 8.34 (d, *J*= 8.5 Hz, 4H, 4xH$_{3''}$), 8.43 (d, *J*= 8.5 Hz, 4H, 4xH$_{4''}$), 9.89 (s, 2H, 2xH$_{2'}$). **^{13}C-NMR** (125 MHz, DMSO-*d*$_6$): 50.8, 53.9, 113.5, 114.1, 122.8, 123.9, 126.6, 126.7, 130.0, 130.6, 131.6, 138.3,139.1, 143.7, 150.5, 153.1, 190.7.

*General procedure for synthesis of quaternary salts **5a-g** under conventional TH*

A solution of 2,6-*bis*((1*H*-imidazol-1-*yl*)methyl)pyridine **3a** (1 mmol, 1 equiv., 0.24 g, dissolved in 40 mL acetone using the ultrasound bath) and the *α*-halogeno- ester and amide (2.4 mmol, 2.4 equiv., 0.22 mL **4a**, 0.44 g **4b**) or the bromacetophenone derivatives (2.8 mmol, 2.8 equiv., 0.56 g **4c**, 0.78 g **4d**, 0.65 g **4e**, 0.68 g **4f**, 0.64 g **4g**) solubilized in acetone (15 mL), was magnetically stirred for 24 h, to give the corresponding quaternary salts **5a-g**. The completion of the reactions was carried out using thin layer chromatography. The obtained salts were filtered off, washed 2 times with the same solvent (10 mL) and dried in vacuo. No other purification required.

*General procedure for synthesis of quaternary salts **6a-g** under conventional TH*

To a solution of 2,6-*bis*((1*H*-benzo[*d*]imidazol-1-*yl*)methyl)pyridine **3b** (1 mmol, 1 equiv., 0.34 g, dissolved in 40 mL acetone using the ultrasound bath) were added the *α*-halogeno- ester and amide (4.8 mmol, 4.8 equiv., 0.73 mL **4a**, 0.89 g **4b**) or the bromacetophenone derivatives (4.5 mmol, 4.5 equiv., 0.89 g **4c**, 1.25 g **4d**, 1.05 g **4e**, 1.1 g **4f**, 1.03 g **4g**) solubilized in acetone (20 mL). The solutions were refluxed for 12 h and magnetically stirred for another 12 h, resulting the desired salts **6a-g**. Thin layer chromatography was used for follow the evolution of chemical reactions. The precipitates were collected by filtration, then washed with acetone (3 times with 10 mL) and dried in vacuum. No other purification required.
